# What does it take? Healthcare professional’s perspective on incentives and obstacles related to implementing ICTs in home-based elderly care

**DOI:** 10.1186/1472-6963-14-S2-P45

**Published:** 2014-07-07

**Authors:** Martha Therese Gjestsen, Siri Wiig, Ingelin Testad

**Affiliations:** 1 Centre for Age-Related Medicine, Psychiatric Division, Stavanger University Hospital, Stavanger, Norway; 2 Department of Health Studies, Faculty of Social Sciences, University of Stavanger, Stavanger, Norway

## Background

The purpose of this study is to identify obstacles and incentives associated with implementation of Information and Communication Technologies (ICTs) in elderly home-based care services, from a healthcare professional perspective. The use of ICTs in elderly homecare is heralded as part of the solution to many of the imperatives currently challenging healthcare systems, but deploying new technology and practice innovations in this complex system can be a challenge. There is poor understanding of what healthcare professionals regard as vital when implementing ICTs in elderly homecare, thus the likelihood of successful implementation is threatened.

The following research question guided our study: How do healthcare professionals describe incentives and obstacles related to implementation of ICTs in home-based care for the elderly?

## Materials and methods

Two focus group interviews with 6 participants in each group were conducted. The informants represented home-based care in a Norwegian municipality and were healthcare professionals in direct patient care or nurse managers administrating care services for the elderly. Through content analysis of the transcribed interviews, incentives and obstacles regarding implementation were identified.

## Results

From a healthcare professional perspective the main incentive to implement ICTs was the practical use in daily care; different patient groups were identified where the use of ICTs potentially could increase the elderly persons’ ability to live more independently in their own home for a longer period of time. The potential for a more precise clinical assessment of home-dwelling elderly receiving care services as well as more rational time use for staff were also described as incentives. Obstacles related to implementation where uncertainty about legislation regulating the use of ICTs, concerns about which impact ICTs may have on relationships with patients, and ethical issues related to the use of ICTs in home-based care, such as surveillance and tracking of persons unable to give informed consent.

## Conclusion

This study identified three incentives related to implementation: 1) positive impact on patients’ degree of autonomy and independency; 2) increased efficiency in day-to-day practice and 3) increased effectiveness in clinical assessments. The main obstacles identified were: 1) uncertainty concerning legislation regulating the use of ICTs, 2) ambiguity towards which role ICTs will play in the day-to-day contact with patients and 3) ethical issues related to the use of ICTs in home-based care.

